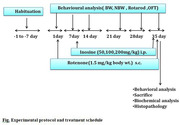# Neuroprotective Effect of Filgrastim against Rotenone induced Parkinson’s disease in Rats

**DOI:** 10.1002/alz.088778

**Published:** 2025-01-09

**Authors:** Hema Chaudhary, Mohit Agrawal

**Affiliations:** ^1^ School of Medical & Allied Sciences, K.R. Mangalam University, Gurugram, Haryana India

## Abstract

**Background:**

Parkinson’s disease is an hypokinetic disorder characterized by selective loss of dopaminergic in substantia nigra pars compacta (SNPc) region of mid‐brain. Dopaminergic degeneration of neurons is considered to be due to oxidative stress, neuroinflammation, neurons mitochondrial dysfunction and glutamate excitotoxicity etc. Filgrastim has been reported to produce anti‐oxidant, anti‐inflammatory and neuromodulatory actions in previous studies.

**Method:**

In the current study we have investigated the role of filgrastim against rotenone induced motor deficit and biochemical abnormalities in Wistar rats. Rats were treated with 1.5mg/kg rotenone subcutaneously for 35 days. filgrastim (50,100 and 200 mg/kg i.p.) was administered after 30 minutes of rotenone administration from day 7^th^ to 35^th^ day. Rotenone caused significant reduction in motor functions and body weight and produced elevation in striatal oxidative burden. The biochemical analysis revealed that there is reduction in the level of anti‐oxidants (GSH, Catalase, and SOD) in the animals following rotenone administration.

**Result:**

Whereas filgrastim treated rats were stable and regained their body weight. In addition filgrastim significantly attenuated rotenone induced motor deficit and striatal oxidative stress.

**Conclusion:**

Outcomes of the current study suggest neuroprotective potential of filgrastim and its ability to correct movement disability and thus could prove to be useful candidate molecule in the management of motor disorders.